# The Relationship between Environmental Regulations and Green Economic Efficiency: A Study Based on the Provinces in China

**DOI:** 10.3390/ijerph18030889

**Published:** 2021-01-20

**Authors:** Gongli Luo, Xiaotong Wang, Lu Wang, Yanlu Guo

**Affiliations:** College of Economics and Management, Shandong University of Science and Technology, Qingdao 266590, China; luogongli1112@126.com (G.L.); zlxiaotong@163.com (X.W.); gylsdust@163.com (Y.G.)

**Keywords:** environmental regulations, green economic efficiency, super-efficiency SBM model with undesirable outputs

## Abstract

This study examined the relationship between environmental regulations (ER) and green economic efficiency (GEE) based on the panel data of 30 provinces in China from 2008 to 2017. Firstly, GEE was calculated and evaluated using the super-efficiency SBM model with undesirable outputs. Secondly, the impact of ER on GEE was studied with the Tobit model. Finally, this article draws conclusions based on the above analysis and offers some suggestions for government and enterprise. The results show that the GEE of China is generally low. The GEE of the eastern region is much higher than that of the middle and western regions, with the western region performing slightly better than the middle. From west to east, there is a V shape, with high efficiency in the west and east and low efficiency in the middle. The impact of ER on GEE has the characteristics of nonlinearity and spatial heterogeneity. At the national level, as well as in the middle and western regions, the impact of ER on GEE shows an inverted U shape that first rises and then falls. ER are currently within the range conducive to the development of GEE. If the intensity of ER exceeds the critical value, they will have a negative impact on GEE. In the eastern region, the impact of ER on GEE is shown as a U shape that first falls and then rises. At present, the ER are not of sufficient intensity to contribute to the improvement of GEE. Only when the intensity of the ER exceeds the critical value will they have a positive influence on the GEE.

## 1. Introduction

The Paris Agreement signed by the 21st Conference of the Parties to the “United Nations Framework Convention on Climate Change” in 2015 stated that all parties would deal with the global threats of climate change and keep the global average temperature rise within 2 °C compared with that before industrialization, striving to keep it within 1.5 °C. In the same year, the Fifth Plenary Session of the 18th Central Committee of the Communist Party of China called for “development that is innovation-driven, coordinated, green, oriented toward global progress, and beneficial to all”. In green economic development, not only the quantity of growth but also the quality of development is crucial. Promoting green growth and implementing the Green New Deal have become a common choice of major economies around the world. Developing green economies and seizing the commanding heights of global competition in the future have become an important national strategy. Green economic development is a means of economic growth and social development that targets economic efficiency, harmony between the economy and environment, and sustainable social development, and it has connotations of resource conservation and environmental friendliness. Improving the efficiency and quality of economic development is a significant way to promote green development. The quality of economic development is based on a comprehensive evaluation of the economic development of a country and region looking beyond total economic output. Under the conditions of limited resources and environmental carrying capacity, improving the efficiency of input is a better path to economic development than relying on economic expansion alone. In the process of economic development, China pays more attention to efficiency and quality, such as urban ecological efficiency, energy and resource allocation efficiency, and total factor productivity, which all reflect the concept of green economic efficiency to a certain extent. Ahmed et al. [1] defined the input–output efficiency of energy consumption and pollutant emissions as green economic efficiency, which, taking resource input and environmental costs into account, is believed to be a crucial indicator for evaluating the production efficiency of a country or region, and it also reflects the efficiency in using natural resources and reducing environmental pressure while pursuing economic benefits. Data envelopment analysis (DEA) as a linear programming method is widely applied for assessing the efficiency and productivity of decision-making units (DMUs). Tao et al. [2] used the DEA-SBM (slacks-based measure) model to measure the efficiency of the provincial green economy in China, and Zhao et al. [3] used the super-efficiency SBM model with undesired outputs to measure the efficiency of the Chinese green economy and analyzed its influencing factors. Wen et al. [4] examined the differences in regional efficiency of China’s construction industry by combining multiregional input–output and DEA methods. In recent years, there has been increased research on the measurement of green economic efficiency, the evolution of the temporal and spatial patterns of such, and its influencing factors.

Environmental regulations, aimed at environmental protection and resource conservation, refer to the government’s direct or indirect control of and intervention in resource utilization by enterprises. Qian and Liu [5] pointed out that the research on the relationship between environmental regulations and economic efficiency in international academia mainly adopts four hypotheses: the constraints hypothesis, environmental competition hypothesis, win–win hypothesis, and uncertainty hypothesis. The constraints hypothesis is that environmental regulations will bring additional burdens on enterprises and adversely affect the economic efficiency of enterprises, departments, or regions. Christainsen and Haveman [6] found that environmental regulations caused the labor productivity growth rate of the US manufacturing sector to decline from 1958 to 1977. The environmental competition hypothesis holds that regions will compete to adopt lower environmental standards to avoid efficiency losses, thereby exacerbating the deterioration of overall environmental quality. Ge et al. [7] found that the efficiency loss caused by the environmental regulations of local and neighboring cities inhibited the inclusive growth of the country and region. The win–win hypothesis, also called the Porter hypothesis, is that the promotion of appropriate environmental regulations may encourage enterprises to improve production efficiency through technological innovation. Domestic enterprises can gain a competitive advantage in the international market, thereby improving industrial efficiency and corporate performance, creating a “win–win” situation regarding environmental protection and economic growth [8,9,10]. The uncertainty hypothesis believes that the uncertain factors surrounding environmental regulations and economic efficiency lead to uncertainty regarding the impact of the former on the latter. The research of Rennings and Rammer [11] supports this hypothesis. There is no consensus regarding the impact of environmental regulations on economic efficiency. Whether the influence of environmental regulations on green economic efficiency corresponds to the above hypothesis requires further studies.

According to the sustainable development strategy, “green” is a necessary condition for sustainable development and the basis for achieving high-quality economic development [12,13]. Against the background of the “new normal” in economic development, there is a general trend of changes in the mode of economic development, promoting industrial transformation and upgrading, and improving the quality of the economy. The traditional method of using a single indicator to measure the level of economic development has certain drawbacks. Since undesirable outputs (such as air pollution and water pollution) have some impact on economic efficiency, it is necessary to build a systematic evaluation system for green economic efficiency to measure economic growth. Based on the above analysis, this article attempts to answer the following questions: What is the current level of China’s green economic efficiency, and are there visible differences in different regions? Does the impact of environmental regulation on green economic efficiency have nonlinear characteristics? Is there any regional heterogeneity in the relationship between the two? Based on the above issues and previous research, this study approached the issue in the following ways. Firstly, green economic efficiency was calculated using the super-SBM model with undesirable outputs and analyzed in different regions. Secondly, the impact of environmental regulations on green economic efficiency was studied with the Tobit model, followed by further studies to test whether the relationship between the two was nonlinear.

## 2. Literature Review and Hypothesis

A review of the literature revealed relatively little on the relationship between environmental regulations and green economic efficiency. Scholars have studied the impact of the former on the latter in terms of energy and economic efficiency, productivity, and ecological efficiency, etc. The improvement of various efficiency would inevitably have varying degrees of influence on green economic efficiency. Regarding the impact of environmental regulations on green economic efficiency, there are three main points of view: the constraint hypothesis, Porter hypothesis, and uncertainty hypothesis.

Supporters of the constraint hypothesis believe that environmental regulations have a negative impact on economic development by increasing costs, etc. Gollop and Roberts [14] found that regulations regarding sulfur dioxide emissions have resulted in both significantly higher costs of generation and markedly lower rates of productivity growth. The increase in production costs would have a potential inhibitory effect on economic development [15], which is not conducive to the improvement of economic efficiency. Yang and Xie [16] concluded that environmental regulations within different ranges of intensity noticeably increased carbon emissions by restraining technological innovation. Li et al. [17,18] found that energy efficiency, energy use, economic growth, and clean energy substitution exert a distorting influence on CO_2_ emissions that contributes to increased CO_2_ emissions in Nigeria. However, environmental diplomacy has been demonstrated to reduce CO_2_ emissions for developing countries in the short term. Wu et al. [19] found that direct government supervision has a negative impact on heterogeneous innovation investment. Moreover, under some conditions, strict regulations could cause firms to be reluctant to innovate and to produce a single standard product rather than distinct products for different market segments [20]. Generally speaking, the dampened enthusiasm of enterprises for innovation would lead to slowing technological progress, hindering productivity growth, which is not conducive to reducing pollution emissions, thus negatively affecting the efficiency of the green economy. Due to the time lag of policies, old environmental policies were found to be unable to promote the sustainable growth of green total factor productivity and to induce enterprises to increase polluting economic outputs in order to compensate for the cost of reducing emissions, thereby worsening the environmental situation [21]. High-pollution economic output is contrary to the economic development model entailing low pollution and energy consumption pursued in contemporary economic development, instead promoting the deterioration of environmental conditions and hindering the improvement of green economic efficiency.

Supporters of the Porter hypothesis believe that environmental regulations have a positive impact on economic development through stimulating innovation and other means. Strict environmental regulations have been found to not only reduce pollution but also be conducive to the innovation of manufacturing enterprises, increasing the number of green patents and enhancing their quality [22]. Kim and Brown [23] found domestic demand-pull and technology-push policies to positively affect domestic lighting patenting. Yang and Song [24] found that environmental regulations could indirectly break the resource curse by improving green technology innovation, reducing resource dependence, and promoting progress in manufacturing, helping the development of the green economy. Thus, environmental regulations may promote green economic efficiency to a certain extent. Environmental regulations in different fields have been proven useful for reducing wastewater and solid-waste emissions, improving air quality, and reducing carbon emissions [25,26,27]. The reduction of pollutants such as wastewater, waste gas, and solid waste accordingly reduces the undesirable outputs that hinder the improvement of green economic efficiency. Therefore, reducing pollution emissions is an important step in promoting the development of green economic efficiency. Furthermore, environmental regulations can improve total factor productivity by promoting research and development (R&D) investment and encouraging technological innovation [28,29]. Ciocci and Pecht [30] believed that environmentally motivated changes were beneficial in terms of waste and emission reduction, helping to improve energy and economic efficiency. The improvement of productivity, energy efficiency, and economic efficiency promoted resource conservation to a certain extent and enhanced the efficiency of the green economy. Some scholars in China have studied the direct and moderating effects of environmental regulations on green economic efficiency. The study results of He and An [31] show that environmental regulations are conducive to the improvement of green development efficiency, protecting the environment, and promoting high-quality economic development. Zhang and Qin [32] found that environmental regulations and local green efficiency were significantly positively correlated. The rationalization and the optimization of the industrial structure have considerably promoted the improvement of regional green efficiency. This implies that environmental regulations have had an indirect positive impact on green economic efficiency by promoting industrial transformation and the upgrading and changing of production patterns.

Supporters of the uncertainty hypothesis believe that the impact of environmental regulations on economic development is difficult to predict. Kuosmanen et al. [33] believe that the influence of environmental policies on the economy is time-dependent: in the short term, the positive effects of environmental policies on the economy are difficult to ascertain, while in the long term, there are remarkable economic benefits. The changes in economic efficiency inevitably influence green economic efficiency. The impacts of different types of environmental regulations are heterogeneous. Market-incentive environmental regulations have improved total factor environmental governance efficiency in the short term, but a further increase in intensity inhibited total factor environmental governance efficiency [34]. A U-shaped relationship between cost-based environmental regulations and the green productivity of industry has been found [35]. Evidence from China shows that the impact of environmental regulations on green productivity growth has nonlinear characteristics, the relationship between the two presenting an inverted U shape. If the strict intensity of environmental policy is lower than 3.08, it has a positive impact on the growth of green productivity. Otherwise, the impact becomes negative [36]. Hu and Liu [37] concluded that the impact of environmental regulations on green economic efficiency presents an inverted U shape that first rises and then falls. Studies have revealed that the transformation and upgrading of industrial structure has affected green economic efficiency. Since the impact of environmental regulations on the green transformation of industry is uncertain, the effect of the former on green economic efficiency may be uncertain to a certain extent.

In summary, some scholars have researched the economic and environmental consequences of environmental regulations, mostly focusing on the impact of the latter on ecological efficiency, total factor productivity, and high-quality economic development. However, the literature is rather lacking in direct studies of the effect of environmental regulations on green economic efficiency. In terms of the methods for measuring green economic efficiency, most scholars have used the DEA method, but relatively few have used the super-efficiency SBM model with undesirable outputs. Most of the research on environmental regulations and green economic efficiency has focused on the relationship between them, while there is relatively little literature on the spatial and temporal distribution of green economic efficiency. Therefore, green economic efficiency was calculated by using the super-SBM model with undesirable outputs in this study, before analyzing the spatial and temporal distribution characteristics of green economic efficiency. We draw an interesting conclusion, that is, the distribution of green economy efficiency in China is V shaped from east to west, instead of the step-like pattern that scholars have found. Furthermore, the model was combined with the Tobit model to empirically test the relationship between environmental regulations and green economic efficiency. This paper enriches the literature on green economic efficiency, helping to clarify the impact of environmental regulations on green economic efficiency, and guiding regions in formulating reasonable policies. Environmental regulations should be established at an appropriate intensity to promote the efficiency of the regional green economy.

## 3. Data and Models

### 3.1. Data

Each Province has a different degree of environmental regulations, and the differences correspond to the objective each Province wants to achieve. According to the research purposes and availability of relevant data, this study empirically analyzed the relationship between environmental regulations and green economic efficiency by using the data of 30 provinces and cities in China (excluding Hong Kong, Macau, Taiwan, and Tibet) for 2008–2017. The data used for examination are from the China Statistical Yearbook, China Environment Yearbook, and China Economic Net Statistical Database. Green economic efficiency was calculated using MaxDEA 7.0 and including input–output variables in the super-SBM model with undesirable outputs. The relationship between environmental regulations and green economic efficiency was analyzed using Stata 15.0, including the panel data in the Tobit model.

### 3.2. Green Economic Efficiency Measurement Model

#### 3.2.1. Variable Selection

The selection of indicators directly affects the reliability of the efficiency value. Green economic efficiency is the comprehensive economic efficiency after comprehensively considering the input of production factors, resource consumption, and environmental costs. Drawing lessons from existing research, the green economic efficiency index system constructed in this article is shown in Table 1. The input and output variables that need to be considered in calculating the efficiency of the green economy are as follows:

##### Investment Indicators

Depending on the production function, labor and capital input is the fundamental input of production factors. Energy input is an important part of production input, and the energy industry is an important part of basic industry. More investment in the energy industry means more energy such as coal, oil, and electricity are consumed for production. Employers in urban units in various regions represent the labor input. Fixed asset investment across the whole of society represents capital expenditure. Energy industry investment represents energy consumption.

##### Desirable Output Indicators

Generally, the desirable output is the gross domestic product, regional gross domestic product, or industrial added value. This study took the gross regional domestic product as the desirable output.

##### Undesirable Output Indicators

Economic development is usually accompanied by problems such as environmental pollution. Universal undesirable outputs include wastewater, exhaust gas, and solid-waste discharge, which are represented by industrial wastewater discharge, sulfur dioxide discharge, and general industrial solid-waste generation in this study.

#### 3.2.2. Super-SBM Model with Undesirable Outputs

Data envelopment analysis (DEA) is a linear programming method for assessing the efficiency and productivity of units called decision-making units (DMUs). The traditional DEA model ignores the slackness of the input and output, making the measurement of efficiency inaccurate. Tone subsequently proposed the slacks-based measure (SBM) model, the slacks-based measure of super-efficiency (super-SBM) model, and the SBM model to deal with undesirable outputs [38,39,40]. The SBM model takes slack variables into account. As the slack degree of input and output indicators changes, the efficiency value is adjusted accordingly to make the results more accurate. Super-SBM is a type of super-efficiency DEA model that combines super-efficiency and the SBM model. It solves the problem of the efficiency values of multiple DMUs equaling 1 when the efficiency is measured. It can also determine and rank efficient DMUs. The SBM model with undesirable outputs may cause multiple DMUs to be effective at the same time, which is not conducive to sorting the DMUs. Therefore, this study used the super-SBM model with undesirable outputs for evaluating green economic efficiency.

Assuming that the production system has *n* DMUs, each province (region, city) is considered a DMU, consisting of three vectors: the input, desirable output, and undesirable outputs. These three vectors are, respectively, $x\in R^{m},y_{g}\in R^{s_{1}},\;\mathrm{and}\;y_{b}\in R^{s_{2}}$. The definable matrix *X*, $Y^{g},\mathrm{and}\;Y^{b}$ is as follows:

(1) $$X=\left[x_{1},x_{2},\ldots ,x_{n}\right]\in R^{m\times n}>0,Y^{g}=\left[y_{1}^{g},y_{2}^{g},\ldots ,y_{n}^{g}\right]\in R^{s_{1}\times n}>0,Y^{b}=\left[y_{1}^{b},y_{2}^{b},\ldots ,y_{n}^{b}\right]\in R^{s_{2}\times n}>0$$

Referring to the practice of Tone [39] and Li et al. [41], a limited possible production set that excludes the decision-making unit (*x*_0_, *y*_0_) is:

(2) $$P\backslash \left(x_{0},y_{0}\right)=\{\left(x,{\bar{y}}^{g},{\bar{y}}^{b}\right)|\bar{x}\geq \overset{n}{\underset{j=1}{\sum }}\tau_{j}x_{j},{\bar{y}}^{g}\leq \overset{n}{\underset{j=1}{\sum }}\tau_{j}y_{j}^{g},{\bar{y}}^{b}\geq \overset{n}{\underset{j=1}{\sum }}\tau_{j}y_{j}^{b},{\bar{y}}^{g}\geq 0,\tau \geq 0\}$$

The super-SBM model with undesirable outputs (VRS) is as follows:

(3) $$\theta^{*}=min\frac{\frac{1}{m}\sum_{i=1}^{m}{\bar{x}}_{i}/x_{i0}}{\frac{1}{s_{1}+s_{2}}\left(\sum_{r=1}^{s_{1}}{\bar{y}}_{r}^{g}/y_{r0}^{g}+\sum_{l=1}^{s_{2}}{\bar{y}}_{l}^{b}/y_{l0}^{g}\right)}\;s.t.\{\begin{array}{c} \bar{x}\geq \sum_{j=1,\neq 0}^{n}\tau_{j}x_{j}\\ {\bar{y}}^{g}\leq \sum_{j=1,\neq 0}^{n}\tau_{j}y_{j}^{g}\\ {\bar{y}}^{b}\leq \sum_{j=1,\neq 0}^{n}\tau_{j}y_{j}^{b}\\ \bar{x}\geq x_{0},{\bar{y}}^{g}\leq y_{0}^{g},{\bar{y}}^{b}\geq y_{0}^{b},\;{\bar{y}}^{g}\geq 0,\tau \geq 0,\sum_{j=1,\neq 0}^{n}\tau_{j}=1 \end{array}$$

where θ* is the target efficiency value. $x,\;y^{g},\;\mathrm{and}\;y^{b}$ are the input, desirable output, and undesirable outputs, respectively. *s*^−^, *s^g^*, and *s^b^* are the slack in the input, desirable output, and undesirable outputs. *τ* is the weight vector. $\sum_{j=1,\neq 0}^{n}\tau_{j}=1$ represents the variable returns to scale. The larger the θ*, the greater the efficiency.

### 3.3. Regression Model

#### 3.3.1. Variable Selection

##### Explained Variable

The explained variable in this study is green economic efficiency (GEE), which was calculated through the super-efficiency SBM model with undesirable outputs.

##### Explanatory Variable

The explanatory variable in this study is the intensity of environmental regulations (ER), which was measured by the investment in the control of industrial pollution in each region. The greater the investment, the stricter the local government’s control of pollution and the greater the intensity of environmental regulations.

##### Control Variables

Drawing on the research of Su and Zhang [42], the following indicators were selected as control variables. (1) The economic development level (GDP). Social and economic development contributes to the improvement of regional green economic efficiency. The GDP per capita of each province was used to measure the development of the economy. (2) The industrial structure (IND). Secondary industry is the industrial sector that processes raw materials. Pollutants generated during processing are a crucial source of undesirable outputs, which usually inhibit the efficiency of the green economy. Therefore, the added value of secondary industry was used to measure the industrial structure. (3) The level of fixed assets (FA). Fixed assets can expand the space for economic development and increase the fixed asset investment of the whole of society with great significance. Therefore, the fixed asset investment across the whole of society as a proportion of regional GDP was used to measure the level of fixed assets. (4) The level of openness to the outside world (OPEN). Generally speaking, opening to the outside world is conducive to driving regional economic development, so the total import and export volumes of each region were used to measure the level of opening up. (5) The urbanization level (URB). The urbanization level has an impact on the development of a city’s green economy to a certain degree. Therefore, the proportion of the total population that was urban was taken as a measure of urbanization. This study addressed the above-mentioned variables (except green economic efficiency) logarithmically to eliminate the possible heteroscedasticity problem.

#### 3.3.2. Tobit Model

Since the value of green economic efficiency tends to be between 0 and 1, Tobit model was chosen to test the impact of environmental regulations on green economic efficiency. In addition to the regression analysis of full samples, 30 provinces were divided into eastern, middle, and western regions for testing. Due to the different levels of economic development and intensity of environmental regulation, the impact of environmental regulations on the efficiency of the green economy has uncertain nonlinear characteristics. The square term of environmental regulations was thus incorporated into the measurement model.

The constructed model is as follows:

(4) $${\mathrm{GEE}}_{i,t}=\alpha_{0}+\beta_{1}{\mathrm{ER}}_{i,t}^{2}+\beta_{2}{\mathrm{ER}}_{i,t}+\beta_{3}{\mathrm{GDP}}_{i,t}+\beta_{4}{\mathrm{IND}}_{i,t}+\beta_{5}{\mathrm{FA}}_{i,t}+\beta_{6}{\mathrm{OPEN}}_{i,t}+\beta_{7}{\mathrm{URB}}_{i,t}+\mu_{i,t}+\varepsilon_{i,t}$$

where *i* means a province (*i* = 1, 2,..., 30), t means time, GEE means the green economic efficiency, EG means environmental regulations, ER^2^ means the square term of environmental regulation, GDP means the development level of the economy, IND means the industrial structure, FA means the level of fixed assets across the whole of society, OPEN means the level of opening up, URB means the urbanization level, *μ* means the individual effect used to control individual effects, and *ε* is the error term. If *β*_1_ is significantly positive, there is a U-shaped relationship between environmental regulations and green economic efficiency. If *β*_1_ is significantly negative, there is an inverted-U-shaped relationship between environmental regulations and green economic efficiency. If *β*_1_ is not significant, it indicates that there is no nonlinear relationship between environmental regulation and green economic efficiency.

#### 3.3.3. Calculation of the Critical Value

If there is a nonlinear relationship between environmental regulations and green economic efficiency, it is necessary to calculate the point at which the direction of influence changes. Therefore, the extreme value theorem was chosen in this study to determine the critical value of environmental regulations regarding green economic efficiency. According to the definition of extreme points, suppose that the function f(*x*) is defined in interval I, and *x*_0_ is a point in interval I. If ∃δ > 0, $\forall x\in \left(x_{0}-\delta ,\;x_{0}+\delta \right)$, and f(*x*) > f(*x*_0_) (or f(*x*) < f(*x*_0_)) is satisfied, then f(*x*_0_) is the minimum value (or maximum value) of the function f(*x*), and *x*_0_ is called the minimum (or maximum) point of a function. The necessary and sufficient conditions for the existence of extreme values are as follows:

**Theorem** **1.** *If f(x) is derivable at point x_0_, and x_0_ is the extreme point of function f(x), then f ‘(x) = 0.*

##### The First Derivative Test

Let f(*x*) be a derivable function with f ‘(*x*_0_) = 0; then:

(1) If f ‘(*x*) changes from positive to negative, then f(*x*) has a relative maximum at x_0_.
(2) If f ‘(*x*) changes from negative to positive, then f(*x*) has a relative minimum at x_0_.
(3) If f ‘(*x*) does not change sign at *x*_0_, then f(*x*) has neither a maximum nor a minimum at *x*_0_.

Assume that green economic efficiency (GEE) is a quadratic function of the environmental regulations (ER), satisfying $\mathrm{GEE}\left(\mathrm{ER}\right)=\alpha {\mathrm{ER}}^{2}+\beta \mathrm{ER}+c$. Let $\frac{\partial \mathrm{GEE}(\mathrm{ER})}{\partial \mathrm{ER}}=0$ to solve ${\mathrm{ER}}_{0}$. Substitute ER_1_ and ER_2_ on both sides of ${\mathrm{ER}}_{0}$ into $\frac{\partial \mathrm{GEE}(\mathrm{ER})}{\partial \mathrm{ER}}=2\mathrm{ER}+\beta$. If $\frac{\partial \mathrm{GEE}({\mathrm{ER}}_{1})}{\partial {\mathrm{ER}}_{1}}\times \frac{\partial \mathrm{GEE}({\mathrm{ER}}_{2})}{\partial {\mathrm{ER}}_{2}}<0$, it means that the image direction of the function GEE (ER) has changed. As previously stated, the impact of environmental regulations on green economic efficiency changes at point ${\mathrm{ER}}_{0}$; ${\mathrm{ER}}_{0}$ is the critical value at which the intensity of environmental regulations affects the efficiency of the green economy.

## 4. Empirical Test and Results

### 4.1. Evaluation of Green Economic Efficiency

Green economic efficiency values were obtained for 2008–2017 in China (see Table 2). The average green economic efficiency at the national level was 0.586, widely varying in each province. The maximum value appeared for Qinghai (1.435), while the minimum was for Xinjiang (0.326). There are seven regions with green economic efficiency values greater than 1, accounting for about 23% of the total sample. There are eight provinces with green economic efficiency values lower than 0.5, accounting for 60%. This shows that China’s green economic efficiency is mostly low. Tao et al. [2] have found that not only the whole green economic efficiency is low in China, but also there are large differences among regions. Our analysis is similar to it. There are eight provinces of which green economic efficiency reaches 1, followed by Qinghai (1.435), Beijing (1.193), Shanghai (1.107), Tianjin (1.099), Guangdong (1.093), Jiangsu (1.084), Shandong (1.038), and Hainan (1.00). On the contrary, the bottom five of green economic efficiency averages are Xinjiang (0.326), Yunnan (0.332), Shanxi (0.360), Sichuan (0.362), and Guizhou (0.377).

**Figure 1:**
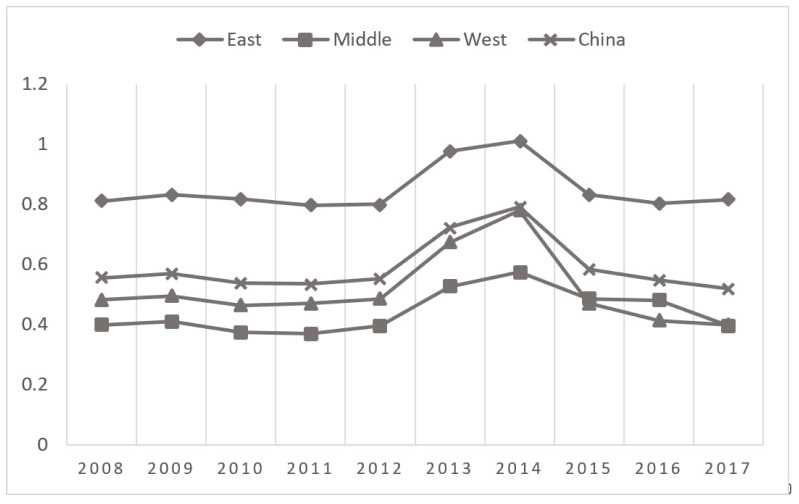
The development trend of green economic efficiency from 2008 to 2017.

The temporal distribution of green economic efficiency (see Figure 1) shows an upward trend from 2008 to 2014, with relative stability from 2008 to 2012 and then remarkable growth from 2012 to 2014. In particular, the growth rate between 2012 and 2013 was rapid, reaching its maximum in 2014. From 2014 to 2015, the decline was relatively large, and then, growth remained stable. Although the changes are inconspicuous, the overall trend is downward. The development of enterprises relied more on high-input, high-energy, and highly polluting production models before 2012, which brought a series of problems such as environmental pollution and resource waste. Therefore, the efficiency of the green economy was generally low. In 2012, the 18th National Congress of the Communist Party of China emphasized the vigorous promotion of the construction of ecological civilization, to reverse the deterioration of the ecological environment. It advocated the comprehensive promotion of resource conservation, to strengthen natural ecosystems, environmental protection, and the construction of ecological civilization systems. In response to the policies, enterprises achieved significant improvements in resource utilization and pollutant emissions. Therefore, the efficiency of the green economy was greatly enhanced between 2012 and 2014. Since 2014, the economic growth of China has slowed down and remained relatively stable. The efficiency of the green economy therefore declined after 2014, but the decline has been inconspicuous in recent years. The efficiency of the green economy in Hainan is always equal to 1 (see Table 2). This is a consequence by Hainan’s economic development model, which mainly relies on the tourism and service industries. This allows it to achieve a relatively large economic output with less input. Its industry is mainly light industry, with less energy input and, correspondingly, fewer pollutant emissions; its green economic efficiency thus remains relatively stable. Compared with 2008, there are 18 provinces with negative growth in green economic efficiency, while 11 are positive. Inner Mongolia, Gansu, and Yunnan are the top three by the margin of decrease, with decreases of 62.95%, 30.29%, and 24.65%, respectively. Ningxia, Hunan, and Liaoning are the top three by the margin of increase, with increases of 102.57%, 48.44%, and 29.89%. The rise of Ningxia was particularly prominent.

**Figure 2:**
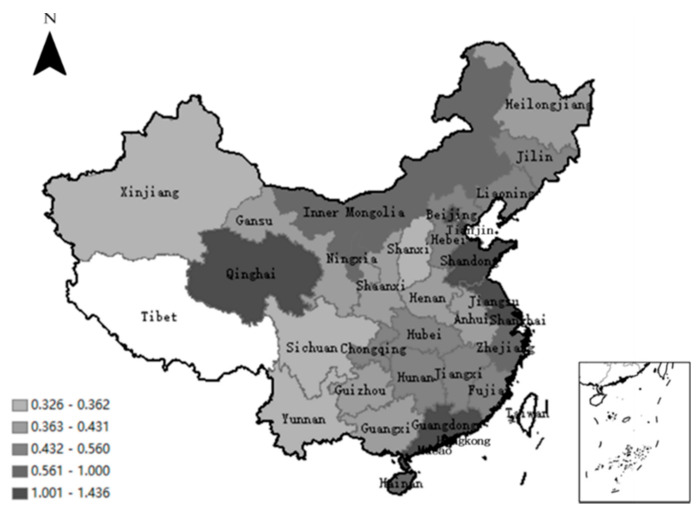
Spatial distribution of average green economic efficiency from 2008 to 2017.

In terms of the spatial distribution of green economic efficiency (see Figure 2), the eastern region is, overall, higher than the national average, while the eastern and western regions are higher than the middle. The green economic efficiency of the three regions from west to the east shows a V shape, with high values on both sides and low in the middle. In terms of rank by green economic efficiency, seven of the top 10 are located in the eastern part, while seven of the bottom 10 are in the western region. All the provinces (except Qinghai) with green economic efficiency greater than 1 belong to coastal areas distributed in the Yangtze River Delta, the Pearl River Delta, and the Bohai Rim economic circle (see Figure 2). Compared with the middle and western inland areas, the coastal areas have higher economic activity and technological innovation capabilities, greater population densities, and a more reasonable industrial structure. The three economic circles are magnets for outstanding talent, the leaders in domestic technological innovation. Their economic development mainly depends on knowledge-intensive industries. Therefore, the green economic efficiency of the eastern coastal areas is, overall, higher than that of the middle and western parts. The midwestern regions are still dependent on traditional agriculture and industry, and their economic development mainly relies on resource-intensive industry. The economic development model of high investment, high pollution, and high energy consumption under the traditional industrialization model is no longer suitable for the theme of green development today, which is to take the path of low resource consumption, low environmental pollution, and sustainable development. The provinces with lower green economic efficiency such as Xinjiang, Yunnan, Shanxi, Sichuan, and others who are developing resource-intensive industries and have abundant resources are particularly problematic. A higher input of energy resources is often concomitant with higher pollution and emissions. Therefore, the green economic efficiency of midwestern regions is, overall, low.

### 4.2. Regression Analysis

#### 4.2.1. The Impact of Environmental Regulations on Green Economic Efficiency

The regression results for environmental regulations and green economic efficiency (see Table 3) show that the nonlinear relationship between ER and GEE has regional heterogeneity. At the national level, the coefficient of regression between ER^2^ and GEE is significantly negative, while that between ER and GEE is positive. It shows that the relationship between environmental regulations and green economic efficiency is an inverted U shape that first rises and then falls. There is a critical value for the impact of environmental regulations on green economic efficiency. The intensity of environmental regulations is currently on the left side of the maximum, which means that further environmental regulations could promote green economic efficiency. The above results further confirm the view of Porter and Van der Linde [43] that the benefits for the environment and economy can result in a win–win situation. Appropriate environmental regulations can reduce undesirable outputs by stimulating innovation, reducing production costs, improving resource utilization, and reducing pollutant emissions, thereby improving the efficiency of the green economy. If the intensity of environmental regulations exceeds the critical value, it has a negative impact on green economic efficiency. Under some circumstances, strict environmental regulations undermine the enthusiasm of enterprises for innovation, leading to an unreasonable industrial structure, increasing potential production costs and reducing the efficiency of the green economy. The conclusion that the impact of environmental regulations on the efficiency of the green economy presents an inverted U shape, first promoting and then hindering, is consistent with the research of the domestic scholars Zhang et al. [44].

In terms of the three major regions, the impact of environmental regulations on green economic efficiency in the midwestern region is consistent with the effects at the national level. The regression coefficient for ER^2^ and GEE is significantly negative, while that for ER and GEE is significantly positive. This implies that the relationship between environmental regulations and green economic efficiency in the middle and western regions is an inverted U shape that first rises and then falls. Environmental regulations currently have a positive effect on green economic efficiency. If the intensity of environmental regulations exceeds the critical value, the impact will turn negative. The relationship between environmental regulations and green economic efficiency in the east is the opposite to that observed in the midwestern area and at the national level. This is because, in the long term, this region retains strong potential in terms of technological innovation and capital investment, enough to withstand the impact of regulations and utilize them as a means of strengthening industrial competitiveness. The regression coefficient for ER^2^ and GEE is significantly positive, while that for environmental regulations and green economic efficiency is significantly negative. Therefore, the impact of environmental regulations on green economic efficiency presents a U shape. Environmental regulations adversely affect green economic efficiency at present. Only when the intensity of environmental regulations exceeds the critical value will they improve green economic efficiency. In the eastern region, the impact of environmental regulations on green economic efficiency shows a U shape that first falls and then rises, consistent with the research conclusions of Su and Zhang [42].

The impact of control variables on green economic efficiency also shows regional heterogeneity. At the national level, the economic development level has a significant positive effect on the efficiency of the green economy in each area. The industrial structure and level of fixed investment assets have a significant negative impact on the efficiency of the green economy. Generally speaking, the higher the proportion of secondary industry, the greater the possibility of increased energy consumption and pollutant emissions. The latter are crucial input and output indicators for measuring green economic efficiency. Therefore, it is generally believed that the larger the proportion of secondary industry in terms of GDP, the greater the number of pollutants discharged from it [45,46,47]. In terms of the three regions, the level of economic development has a positive effect on green economic efficiency, but this is only significant in the western region. The industrial structure has a significant negative effect on green economic efficiency in the eastern part, but an effect not significant in the midwestern region. The fixed assets level has a significant negative effect on green economic efficiency in the western region, but not in the middle and eastern regions. Opening to the outside world has a significant positive effect on green economic efficiency in the east of China, a negative effect in the western part, and an insignificantly positive impact in the middle. The level of urbanization has no significant effect on green economic efficiency in the three regions.

#### 4.2.2. Determination of Critical Value of Environmental Regulations

Considering the research’s purpose and feasibility, the control variable *X_i_* was assumed to be a linear function of the environmental regulations (ER):

(5) $$X_{i}{=k}_{i}{\mathrm{ER}+b}_{i},\;\left(i=3,4,\ldots 7\right)$$

*X*_3_–*X*_7_, respectively, represent the economic development level, industrial structure, fixed asset investment level, opening-up level, and urbanization level. *k_i_* represents correlation coefficient. *b_i_* represents constants. Regression to Equation (5) produces the following results for *k_i_* and *b_i_* (see Table 4):

Substituting Equation (5) into Equation (4) gives:

(6) $$\mathrm{GEE}\left(\mathrm{ER}\right)=\beta_{1}\times {\mathrm{ER}}^{2}+\beta_{2}\times \mathrm{ER}+\overset{7}{\underset{i=3}{\sum }}\beta_{i}\left(k_{i}{\mathrm{ER}+b}_{i}\right)+c_{i}\left(i=1,2,\ldots 7\right)\;$$

Letting the first derivative of Equation (6) be equal to 0, we get:

(7) $$\frac{\partial \mathrm{GEE}\left(\mathrm{ER}\right)}{\partial \mathrm{ER}}=2\beta_{1}\mathrm{ER}+\beta_{2}+\overset{7}{\underset{i=3}{\sum }}\beta_{i}k_{i}=0\;$$

(8) $${\mathrm{ER}}_{0}=-(\beta_{2}+\overset{7}{\underset{i=3}{\sum }}\beta_{i}k_{i})/2\beta_{1}$$

Take ER_1_ and ER_2_ arbitrarily (ER_1_ < ER_0_ < ER_2_), and satisfy the condition of $\frac{\partial \mathrm{GEE}({\mathrm{ER}}_{1})}{\partial {\mathrm{ER}}_{1}}\times \frac{\partial \mathrm{GEE}({\mathrm{ER}}_{2})}{\partial {\mathrm{ER}}_{2}}<0$, which indicates that ER_0_ is the critical value of the intensity of environmental regulations affecting the efficiency of the green economy, and the direction of influence changes at ER_0_. Substituting the data in Table 3 and Table 4 into Equation (8), the critical values of environmental regulations at the national level and in the eastern, middle, and western regions are 7.42, 11.55, 11.84, and 8.59, respectively. At the national level, environmental regulations are, within a range, currently conducive to the development of green economic efficiency. The positive effect on green economic efficiency is optimal when the intensity of environmental regulations is 7.42. If the intensity of environmental regulations exceeds 7.42, it has a negative impact. In the eastern region, environmental regulations do not currently contribute to the improvement of green economic efficiency. Only when the intensity of environmental regulations exceeds 11.55 will it have a positive impact on green economic efficiency. In the middle and western regions, environmental regulations are currently within the range conducive to the development of green economic efficiency. As long as the intensity of environmental regulations in the middle region does not exceed 11.84 and that of those in the western part does not exceed 8.59, they will continue to play a positive role in the efficiency of the green economy.

## 5. Conclusions and Suggestions

### 5.1. Conclusions

Based on the panel data of 30 provinces in China from 2008 to 2017, this study measured China’s green economic efficiency by constructing a super-SBM model with undesirable outputs, analyzing the development trend and spatial distribution characteristics of China’s green economic efficiency in terms of time and space. The results show that the overall efficiency of China’s green economy is relatively low, but a trend of steady change has emerged in recent years. The spatial distribution presents a V shape, with high efficiency in the western and eastern regions and low efficiency in the middle of China.

The impact of environmental regulations on green economic efficiency was empirically tested by constructing a Tobit model. The results show that the impact of environmental regulations on green economic efficiency has the characteristics of nonlinearity and spatial heterogeneity. As far as the national as well as the middle and western regions are concerned, the relationship between environmental regulations and green economic efficiency presents an inverted U shape that first rises and then falls. At present, the intensity of environmental regulations is within the range conducive to green economic efficiency. If the intensity of environmental regulations at the national level exceeds 7.42, that in the middle region exceeds 11.84, and that in the western part exceeds 8.59, the improvement of green economic efficiency will be hindered. Concerning the eastern region, the impact of environmental regulations on green economic efficiency presents a U shape that first falls and then rises. The intensity of environmental regulations is currently insufficient to promote the improvement of green economic efficiency here. Only when the intensity of environmental regulations exceeds 11.55 will an effect be realized. Generally, the level of economic development has a significant positive impact on the efficiency of the regional green economy. The industrial structure and level of fixed asset investment have a significant negative impact on green economic efficiency, while the level of opening up and urbanization have positive-but-not-significant effects.

Although relatively rich conclusions were able to be drawn from the various model tests, the study still has limitations. For example, in the selection of variables in the construction of the green economic efficiency indicator system, energy industry investment was selected as a measure of energy input. Considering that investment in the energy industry varies from traditional fossil fuel facilities to energy conservation improvement or renewable energy production, we should directly use “primary or final energy consumption” to measure energy consumption in the future. According to the First Law of Geography, everything is related to everything else, but things nearby are more related than distant things. Furthermore, the spatial weight matrix could be constructed and the spatial measurement model used to study the spatial correlation of the green economic efficiency of various regions. Further research could strengthen and add to the suggestions provided in this paper, such as specific suggestions for the provinces.

### 5.2. Suggestions

In response to the above conclusions, this article proposes the following recommendations from the perspective of government and enterprises.

Government can first be recommended to strengthen the intensity of environmental regulations and improve the regional environmental protection management system and assessment mechanism. Government departments can appropriately improve the intensity of environmental regulations, strengthen the supervision of pollutant emissions from industrial enterprises, and increase and improve the weight of environmental protection, pollution reduction, and ecological benefits through local performance evaluations. At the same time, incentives and penalties should be introduced in order to encourage innovation, environmental protection, and emission reduction, increasing the enthusiasm of enterprises for R&D innovation and environmental protection. Second, government should adjust the economic structure and promote the transformation and upgrading of the industrial structure. It should continue to advance the optimization and upgrading of industry, making it more reasonable and advanced. Unit energy consumption should be reduced and energy utilization efficiency improved to achieve high-quality economic growth. Increasing the level of opening up, learning and absorbing advanced foreign technology, should be encouraged to promote green economic efficiency. Third, measures ought to be taken to suit local conditions, with importance being attached to regional differences in the intensity of environmental regulations, adhering to the principle of coordinating the division of labor and adopting measures to suit local circumstances. Based on the regional environment, regional advantages should be fully harnessed to develop regional advantage industries, and the intensity of environmental regulations should be adjusted in different regions in a well-timed manner. The primary goal in the eastern region should be to regulate environmental regulations to a level conducive to the efficiency of the green economy. The intensity of environmental regulations that can maximize the efficiency of the green economy should then be determined. Adjusting the environmental regulations to a certain intensity in the middle and western regions could optimize their positive effects on green economic efficiency.

Enterprises would be advised to improve their innovation capabilities and fully embrace the role of the main body for enterprise innovation. They should increase their sense of social responsibility, actively carry out R&D and innovation activities, and improve their technological processes. Enterprises could reduce their production costs and energy consumption, reduce their pollutant emissions, and improve their energy efficiency through new technologies, methods, and means to promote the growth of productivity and green economic efficiency. Second, the economic development model should be transformed and the production pattern of enterprises reformed. The eastern region is vigorously developing emerging technologies such as big data, the Internet of Things, cloud computing, and artificial intelligence to provide technical support for economic development at the national level. The middle and western regions should strengthen the technological transformation of traditional manufacturing industries, actively eliminating outdated production lines with high energy consumption, high pollution, and low output. More attention should be paid to the development of emerging industries such as energy conservation and environmental protection, new energy, and new materials. Third, full use should be made of regional resource advantages to promote high-quality economic development. The eastern coastal areas can play to their advantages in talent, technology, and geographical location, vigorously developing tertiary industries such as the financial and service industries to promote an advanced industrial structure and high-quality economic development. The middle and western regions can play to their rich mineral resources and new energy advantages, actively developing environmental protection industries and introducing innovative technology to traditional mineral industry processing procedures, saving energy, reducing emissions, and promoting green economic development.

## Figures and Tables

**Table 1:** Measurement system for green economic efficiency indicators.

First-Level Indicators	Second-Level Indicators	Third-Level Indicators
Input indicators	Capital investment	Whole society fixed-asset investment
	Labor input	Employees in urban units
	Energy input	Energy industry investment
Output indicators	Desirable output	Gross regional domestic product
	Undesirable outputs	Industrial wastewater discharge
		Sulfur dioxide emissions
		Industrial solid-waste discharge

**Table 2:** Green economic efficiency values in provinces of China from 2008 to 2017.

Regions/Year	2008	2009	2010	2011	2012	2013	2014	2015	2016	2017	Mean	Rank
Beijing	1.169	1.165	1.167	1.150	1.157	1.375	1.167	1.195	1.188	1.208	1.193	2
Tianjin	1.083	1.114	1.107	1.115	1.085	1.116	1.095	1.092	1.092	1.087	1.099	4
Hebei	0.487	0.510	0.451	0.428	0.413	0.552	0.726	0.462	0.406	0.536	0.490	14
Liaoning	0.368	0.415	0.435	0.431	0.461	0.497	1.127	0.582	0.440	0.478	0.496	13
Shanghai	1.033	1.028	1.078	1.089	1.116	1.156	1.074	1.145	1.229	1.136	1.107	3
Jiangsu	1.066	1.087	1.085	1.081	1.087	1.258	1.041	1.057	1.044	1.052	1.084	6
Zhejiang	0.791	0.805	0.839	0.718	0.716	1.003	1.127	0.705	0.727	0.68	0.801	11
Fujian	0.432	0.467	0.394	0.366	0.372	1.124	0.714	0.391	0.370	0.359	0.463	16
Shandong	1.065	1.054	1.036	1.039	1.026	1.020	1.095	1.025	1.017	1.009	1.038	7
Guangdong	1.096	1.099	1.088	1.087	1.094	1.093	1.097	1.095	1.096	1.090	1.093	5
Hainan	1.000	1.000	1.000	1.000	1.000	1.000	1.000	1.000	1.000	1.000	1.000	8
Eastern	0.811	0.833	0.818	0.797	0.800	0.976	1.011	0.832	0.803	0.817	0.847	
Shanxi	0.363	0.328	0.317	0.320	0.289	0.445	1.038	0.252	0.238	0.380	0.360	28
Heilongjiang	0.413	0.401	0.381	0.325	0.327	0.627	0.48	0.370	0.347	0.339	0.393	25
Anhui	0.353	0.400	0.353	0.368	0.397	0.535	1.010	0.422	0.370	0.358	0.431	20
Jiangxi	0.433	0.411	0.379	0.375	0.403	0.323	0.302	1.168	1.262	0.365	0.471	15
Henan	0.396	0.398	0.391	0.383	0.413	0.394	0.455	0.416	0.362	0.337	0.393	24
Jilin	0.358	0.418	0.393	0.434	0.492	0.787	0.529	0.456	0.473	0.376	0.460	17
Hubei	0.435	0.452	0.393	0.378	0.445	0.565	0.538	0.503	0.432	0.438	0.454	18
Hunan	0.448	0.487	0.403	0.387	0.441	0.705	0.604	0.715	1.003	0.665	0.560	12
Middle	0.398	0.410	0.375	0.370	0.396	0.527	0.575	0.486	0.481	0.397	0.437	
Sichuan	0.352	0.342	0.308	0.335	0.373	0.463	0.474	0.354	0.326	0.333	0.362	27
Chongqing	0.393	0.422	0.375	0.381	0.398	1.100	0.642	0.387	0.361	0.387	0.453	19
Guizhou	0.385	0.417	0.380	0.309	0.333	0.465	0.531	0.353	0.330	0.321	0.377	26
Yunnan	0.353	0.366	0.341	0.285	0.290	0.437	0.494	0.298	0.264	0.266	0.332	29
Shaanxi	0.364	0.391	0.388	0.364	0.394	0.662	1.117	0.300	0.280	0.278	0.411	21
Gansu	0.416	0.427	0.387	0.349	0.345	0.361	1.506	0.325	0.291	0.290	0.407	22
Qinghai	1.591	1.516	1.506	1.399	1.409	1.548	1.467	1.359	1.309	1.284	1.435	1
Ningxia	0.584	0.622	0.576	1.099	1.082	1.209	1.082	1.122	1.157	1.183	0.933	9
Xinjiang	0.334	0.317	0.312	0.298	0.298	0.534	0.465	0.273	0.265	0.258	0.326	30
Inner Mongolia	1.031	1.030	1.012	1.041	1.048	1.037	1.026	1.016	0.477	0.382	0.864	10
Guangxi	0.376	0.384	0.338	0.372	0.390	0.504	0.649	0.418	0.370	0.314	0.403	23
Western	0.484	0.495	0.463	0.471	0.487	0.673	0.781	0.470	0.413	0.400	0.503	
China	0.555	0.569	0.539	0.536	0.553	0.723	0.791	0.585	0.549	0.519	0.586	

**Table 3:** Regression results for Tobit model.

Variable	China	East	Middle	West
	GEE	GEE	GEE	GEE
ER^2^	−0.003 **	0.046 ***	−0.069 *	−0.005 **
	(−2.037)	(5.085)	(−1.842)	(−2.089)
ER	0.104 ***	−0.989 ***	1.634 *	0.096 **
	(5.220)	(−4.746)	(1.804)	(2.591)
GDP	0.540 ***	0.180	0.046	0.536 ***
	(5.633)	(1.615)	(0.235)	(2.820)
IND	−0.178 ***	−0.320 ***	0.073	−0.059
	(−4.484)	(−5.722)	(0.557)	(−0.829)
FA	−0.272 ***	0.035	0.154	−0.307 **
	(−3.983)	(0.402)	(1.478)	(−2.158)
OPEN	−0.013	0.254 ***	−0.023	−0.217 ***
	(−0.472)	(5.329)	(−0.297)	(−5.165)
URB	−0.177	−0.193	0.095	0.059
	(−0.784)	(−0.690)	(0.193)	(0.142)
Constant	−4.388 ***	4.619 **	−9.992 *	−3.416
	(−3.737)	(2.515)	(−1.738)	(−1.491)

Notes: T-statistics in parentheses; *** *p* < 0.01, ** *p* < 0.05, * *p* < 0.1. GEE: Green economic efficiency. ER: The intensity of environmental regulations. ER^2^: The square term of environmental regulations. GDP:The economic development level. IND: The industrial structure. FA: The level of fixed assets. OPEN: The level of openness to the outside world. URB: The urbanization level.

**Table 4:** Coefficients and constants between environmental regulations and control variables.

Variable	*X*_3_	*X*_4_	*X*_5_	*X*_6_	*X*_7_
k	−0.1	0.026	−0.053	−0.107	0.094
b	10.692	8.442	0.271	6.522	−1.743

## Data Availability

Publicly available datasets were analyzed in this study. This data can be found here: [http://www.stats.gov.cn], [https://db.cei.cn].
